# Diagnostic accuracy of on-site coronary computed tomography-derived fractional flow reserve in the diagnosis of stable coronary artery disease

**DOI:** 10.1007/s12471-021-01647-7

**Published:** 2021-12-15

**Authors:** J. Peper, J. Schaap, B. J. W. M. Rensing, J. C. Kelder, M. J. Swaans

**Affiliations:** 1 Department of Cardiology, St. Antonius Hospital, Nieuwegein, The Netherlands; 2 Department of Radiology, University Medical Centre Utrecht, Utrecht, The Netherlands; 3 Department of Cardiology, Amphia Hospital, Breda, The Netherlands

**Keywords:** Coronary artery disease, Coronary computed tomography angiography, Fractional flow reserve, Computed tomography-derived fractional flow reserve, On-site computation, Diagnostic performance

## Abstract

**Purpose:**

Invasive fractional flow reserve (FFR), the reference standard for identifying significant coronary artery disease (CAD), can be estimated non-invasively by computed tomography-derived fractional flow reserve (CT-FFR). Commercially available off-site CT-FFR showed improved diagnostic accuracy compared to coronary computed tomography angiography (CCTA) alone. However, the diagnostic performance of this lumped-parameter on-site method is unknown. The aim of this cross-sectional study was to determine the diagnostic accuracy of on-site CT-FFR in patients with suspected CAD.

**Methods:**

A total of 61 patients underwent CCTA and invasive coronary angiography with FFR measured in 88 vessels. Significant CAD was defined as FFR and CT-FFR below 0.80. CCTA with stenosis above 50% was regarded as significant CAD. The diagnostic performance of both CT-FFR and CCTA was assessed using invasive FFR as the reference standard.

**Results:**

Of the 88 vessels included in the analysis, 34 had an FFR of ≤ 0.80. On a per-vessel basis, the sensitivity, specificity, positive predictive value, negative predictive value and accuracy were 91.2%, 81.4%, 93.6%, 75.6% and 85.2% for CT-FFR and were 94.1%, 68.5%, 94.9%, 65.3% and 78.4% for CCTA. The area under the receiver operating characteristic curve was 0.91 and 0.85 for CT-FFR and CCTA, respectively, on a per-vessel basis.

**Conclusion:**

On-site non-invasive FFR derived from CCTA improves diagnostic accuracy compared to CCTA without additional testing and has the potential to be integrated in the current clinical work-up for diagnosing stable CAD.

## What’s new?

- This study demonstrated the feasibility of on-site non-invasive fractional flow reserve (FFR) derived from coronary computed tomography angiography (CCTA) for patients with stable coronary artery disease (CAD).
- On-site CT-FFR shows good diagnostic accuracy, sensitivity and specificity using the reference standard FFR.
- On-site CT-FFR improves diagnostic accuracy compared to CCTA alone without additional radiation and testing. It seems an effective step after a positive or inconclusive CCTA procedure.
- On-site CT-FFR has the potential to be integrated in the current clinical work-up for diagnosing stable CAD.

## Introduction

Wire-based fractional flow reserve (FFR) is generally accepted to be the reference standard for the physiological assessment of lesion-specific ischaemia [1]. FFR, the ratio of maximal pressure distal to a stenosis divided by the pressure proximal to a stenosis, is useful as an additional test to anatomical assessment by invasive coronary angiography (ICA) for the diagnosis of coronary artery disease (CAD) requiring revascularisation [2].

Prior to invasive assessment, non-invasive testing for the detection of CAD in patients with complaints of stable chest pain and a low or intermediate probability of CAD, such as coronary computed tomography angiography (CCTA) using anatomical information, is performed [1]. However, several studies indicate that visual anatomical assessment alone might not be sufficient to identify CAD [3, 4]. The specificity of CCTA is moderate, 61–83%, while its sensitivity is high, 87–99% [5–7]. CCTA tends to overestimate stenosis severity mainly in the presence of calcified plaque, leading to a high proportion of patients without haemodynamically significant CAD unnecessarily undergoing ICA and further treatment.

The diagnostic accuracy of CCTA is based only on anatomical severity; the ability to assess the functional severity is lacking. New non-invasive techniques have been developed to add functional characteristics to the anatomical structure derived from CCTA without changes in imaging protocols, additional radiation or medication [7, 8]. A commercially available algorithm, computed tomography-based FFR (CT-FFR) called HeartFlow FFR_CT_ (HeartFlow, Redwood City, CA, USA), has been developed which uses the principles of computational fluid dynamics (CFD) to simulate invasive FFR [9]. It has been evaluated in multicentre trials demonstrating improved diagnostic accuracy beyond that of CCTA alone [8, 10–12]. Nevertheless, HeartFlow FFR_CT_ is based on a complex algorithm and calculations require supercomputers off-site [9]. This comes with additional costs, a delay in obtaining diagnostic information and data protection concerns. To integrate CT-FFR into the clinical workflow, new algorithms that can be used on-site have been developed which also showed improved diagnostic accuracy compared to CCTA alone [13–21]. These algorithms enable computation of FFR on-site in approximately 25 min, whereas the off-site algorithm has a turnover time of approximately 3–6 h. The preliminary results of on-site CT-FFR seem promising, since it has the potential to reduce the number of unnecessary ICA procedures and can potentially be cost-effective [10, 22–24].

The aim of this cross-sectional study was to determine the diagnostic performance of CT-FFR for the diagnosis of lesion-specific ischaemia using FFR as the reference standard and to compare the diagnostic performance of CT-FFR to that of CCTA.

## Methods

A retrospective cohort was composed of a consecutive series of patients who underwent CCTA and FFR measurement between October 2009 and October 2017. Patients were identified by the hospital declaration codes (DBC, *diagnose behandelcombinatie*) of CCTA (DBC code 085140,085141 and 085042) and wire-based FFR (DBC code 033236 and 039476). Exclusion criteria were as follows: (1) history of surgical revascularisation (coronary artery bypass graft), (2) history of stent implantation (percutaneous coronary intervention), (3) cardiac rhythm other than sinus rhythm, (4) aberrant anatomy, (5) ostial stenosis and (6) insufficient image quality. The CT-FFR analyses were performed while blinded to the FFR measurements. Institutional board on human ethics approval was obtained with a waiver regarding informed consent.

### Coronary computed tomography angiography

CCTA was acquired on a Philips Brilliance 64-slice CT scanner (79%), Philips 256-slice Brilliance iCT scanner (7%) (Philips Medical Systems, Best, The Netherlands), or Siemens SOMATOM dual-source CT scanner (15%) (Siemens Healthcare, Forchheim, Germany) with a prospectively ECG-triggered scan mode. A non-enhanced scan to calculate the Agatston calcium score was performed prior to the CCTA. Additional intravenous metoprolol was administered to achieve a heart rate below 62 bpm. The tube voltage ranged between 100 and 120 kVp depending on the body mass index of the patients and the tube current between 600 and 800 mAs. Sublingual nitroglycerin was administered to all patients before image acquisition. All gated images were triggered at 75% of the R‑R interval and reconstructed with a slice thickness of 0.8 mm.

### Computed tomography-derived fractional flow reserve

Three-dimensional (3D) coronary model segmentation and coronary centreline extraction were performed semi-automatically using a commercially available cardiac application (Comprehensive Cardiac Analysis, IntelliSpace Portal Version 9.0, Philips Medical Systems). The coronary lumen segmentation was reviewed in all patients and corrections were made if needed. The effective luminal diameter stenosis was measured on the coronary model images by identifying the minimum diameter compared to the reference diameter for all stenoses. The segmented coronary model was used as input for the on-site CT-FFR lumped-parameter simulation algorithm prototype (Philips Medical Systems). The lumped-parameter model enables fast individual CFD simulations of blood flow in extended vessel networks [25]. FFR values were computed by simulating the pressures in the aorta and in the coronary arteries during simulated hyperaemia and shown as colour gradients superimposed on the 3D coronary tree [13, 25]. A point estimate of the computed FFR was taken at the lesion of interest, e.g. the most severe stenosis on CCTA proximal to the FFR pressure wire position.

### Reference standard: ICA and FFR

ICA biplane views were acquired from all major coronary arteries on Allura catheterisation equipment (Philips Medical Systems) via femoral or radial artery access. Invasive FFR measurements were acquired for clinical indications or research purposes unrelated to this study using a pressure wire passed beyond the stenosis. The exact location of the wire was recorded. Vessel-based analyses were performed from which diagnostic performance at a per-patient level was determined. To compare and assess the diagnostic performance of CT-FFR and CCTA, the clinical standard of FFR ≤ 0.80 indicating haemodynamically significant stenosis was applied. A patient was considered positive when at least 1 vessel had an FFR value ≤ 0.80. The same threshold (CT-FFR ≤ 0.80 indicating haemodynamically significant stenosis) was applied for CT-FFR, and CCTA was considered significant if a lesion caused ≥ 50% reduction in vessel diameter.

### Statistical analysis

Continuous variables are expressed as mean and standard deviation (SD) or median and interquartile range. Categorical variables are presented as totals and percentages. Diagnostic performance was calculated on a per-vessel and a per-patient basis as sensitivity, specificity, positive predictive value (PPV), negative predictive value (NPV) and accuracy. The diagnostic performance variables were calculated as a simple proportion with a 95% confidence interval. The correlation, differences and diagnostic performance between CT-FFR and wire-based FFR were further assessed using the Pearson correlation coefficient, Bland-Altman plot, receiver operating characteristic curve and its area under the curve (AUC). All statistical analyses were performed using R statistical software (www.r-project.org, version 3.4.2).

## Results

**Fig. 1:**
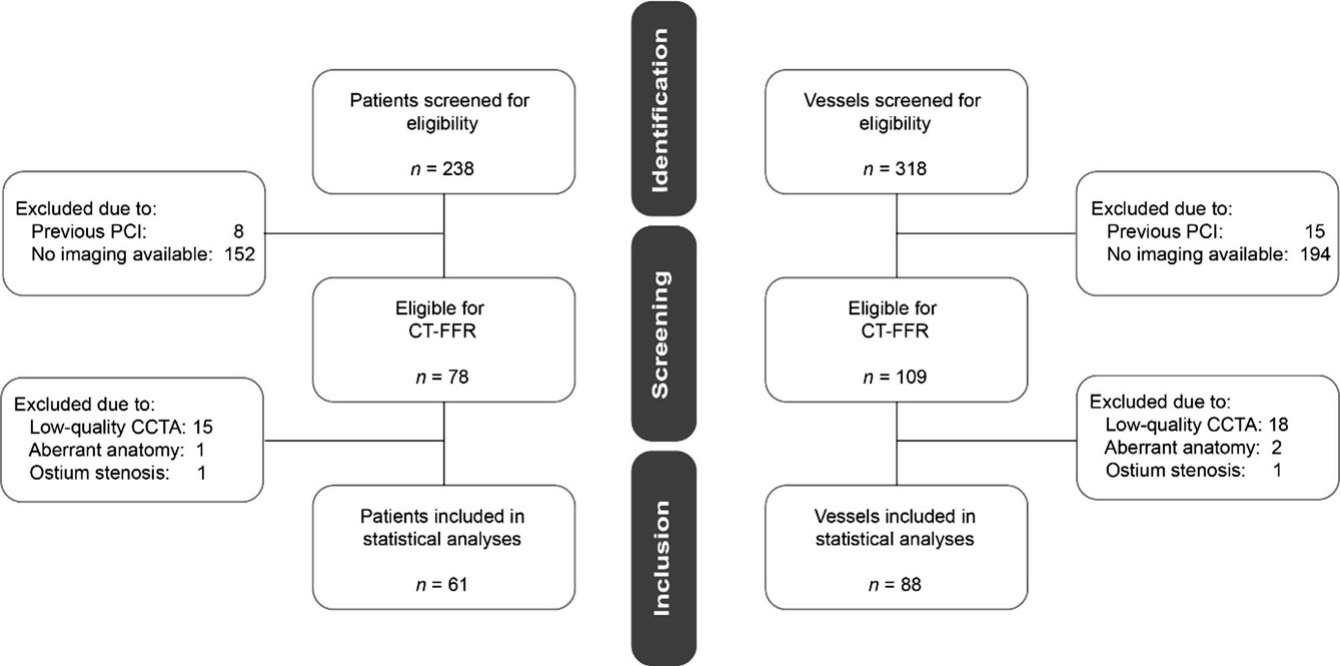
Study enrolment. *CCTA* coronary computed tomography angiography, *CT* computed tomography, *CT-FFR* computed tomography-derived fractional flow reserve, *PCI* percutaneous coronary intervention

**Fig. 2:**
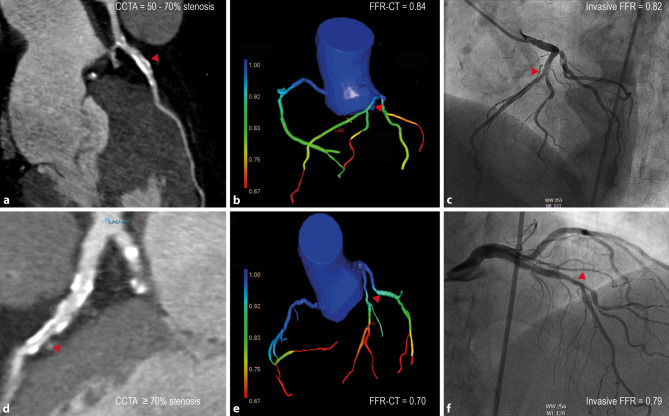
**a**–**f** Example of coronary computed tomography angiography (*CCTA*), computed tomography-derived fractional flow reserve (*CT-FFR*) and invasive FFR in two study patients. **a** CCTA demonstrates a 50–70% obstructive stenosis of the mid-segment of the left anterior descending artery (LAD) and therefore a significant stenosis. **b** The CT-FFR algorithm computes an FFR of 0.84, indicating non-significant vessel ischaemia. **c** Invasive FFR measurement demonstrates obstructive stenosis and an FFR value of 0.82, indicating no vessel ischaemia. **d** The calcified stenosis in the mid-LAD is reduced by more than 70%. **e** CT-FFR indicates the stenosis to be significant with an FFR value of 0.70. **f** Invasive FFR confirms the findings of CCTA and CT-FFR. An FFR of 0.79 is measured, indicating haemodynamically significant coronary artery disease

**Fig. 3:**
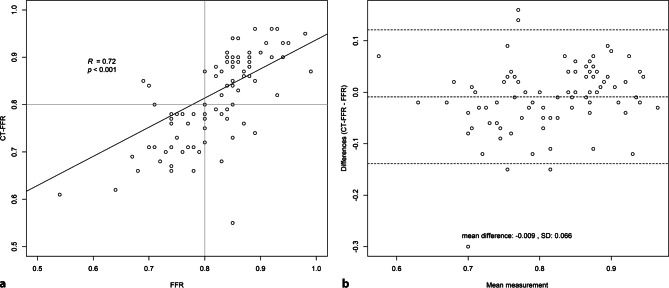
**a**, **b** Scatterplot and per-vessel agreement between computed tomography-derived fractional flow reserve (*CT-FFR*) and FFR. **a** A significant correlation (*r* = 0.72, *p* < 0.001) between CT-FFR and the reference standard FFR is shown. **b** The Bland-Altman plot shows a small bias (mean difference = −0.009) and narrow limits of agreement (SD = 0.066)

**Fig. 4:**
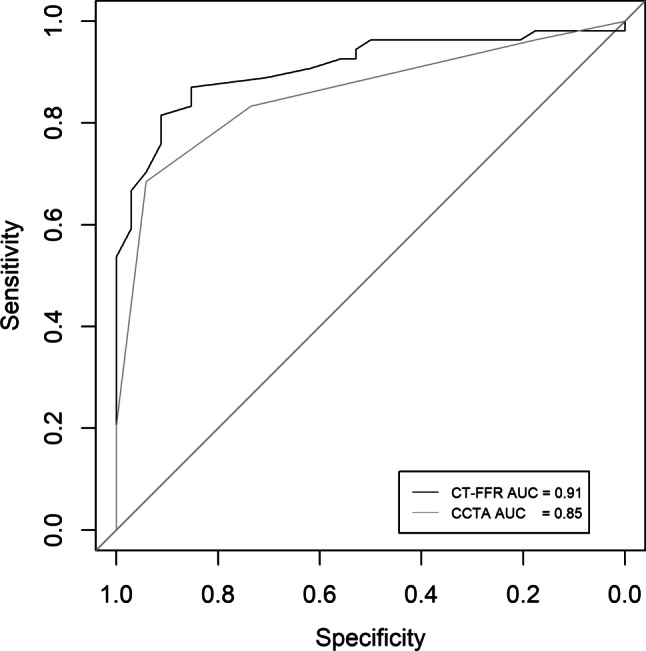
Receiver operating characteristic (ROC) curve of CT-FFR and CCTA. The per-vessel ROC curves for CCTA and CT-FFR. The area under the curve (*AUC*) was not significantly larger (*p* = 0.15) for CT-FFR (AUC = 0.91) than for CCTA (AUC = 0.85)

A total of 238 patients (318 vessels) who underwent CCTA, ICA and FFR measurement were identified as eligible for inclusion in the study (Fig. 1). Patients with a prior history of percutaneous coronary intervention (*n* = 8, 3.4%), without gated CT scan or ICA/FFR (*n* = 152, 63.9%), low-quality CCTA (*n* = 15, 6.3%), aberrant anatomy (*n* = 1, 0.4%) and ostial stenosis (*n* = 1, 0.4%) were excluded. Therefore, 61 patients (88 vessels) were evaluated by CT-FFR and included for statistical analysis (Fig. 2). The patient characteristics and vessel characteristics are provided in Tab. 1. The mean age was 66.0 ± 9.6 years and 46 patients (75.4%) were male. The FFR was haemodynamically significant in 34 (38.6%) vessels and CT-FFR in 41 vessels. The median Agatston score was 317 (CI: 112.3–726.0). The rate of true-positive CT-FFR per vessel was 35.2% (31/88 lesions), true-negative 50.0% (44/88 lesions), false-positive 11.4% (10/88 lesions) and false-negative 3.4% (3/88 lesions). As regards the diagnostic performance of CT-FFR on a per-vessel basis, the sensitivity, specificity, PPV, NPV and accuracy were 91.2%, 81.4%, 93.6%, 75.6% and 85.2%, respectively, while the performance measurements for CCTA were 94.1%, 68.5%, 94.9%, 65.3% and 78.4%. The diagnostic performance of CCTA and CT-FFR on a per-patient level is listed in Tab. 2. There was a good correlation between CT-FFR and FFR, with a Pearson’s correlation coefficient of 0.72 (*p* < 0.001) (Fig. 3). The Bland-Altman plot analysis (Fig. 3) showed a small systematic bias of −0.009 and the limits of agreement were narrow (−0.13; 0.12). The ability of CT-FFR to identify haemodynamically significant CAD seems to be better than that of CCTA (AUC CT-FFR = 0.91 vs AUC CCTA = 0.85, *p*-value = 0.15) (Fig. 4).

**Table 1:** Baseline characteristics. Variables are reported as means ± standard deviation or as frequency (%), unless otherwise specified

Variables (*n* = 61)	Mean ± SD or frequency (%)
*Gender (male)*	46 (75.4)
*Age (years)*	65.98 ± 9.63
*BMI (kg/m*^*2*^*)*	27.41 ± 3.52
*Systolic blood pressure (mm* *Hg)*	135.69 ± 21.82
*Diastolic blood pressure (mm* *Hg)*	82.69 ± 19.19
*Paroxysmal atrial fibrillation*	3 (4.9)
*Smoking*	
No	30 (50.8)
Current smoker	17 (28.8)
Past smoker	12 (20.3)
*Hypertension*	42 (68.9)
*Hyperlipidaemia*	36 (59.0)
*Diabetes*	
No	49 (80.3)
NIDDM	9 (14.8)
IDDM	3 (4.9)
*Family history of CAD*	42 (73.7)
*Creatinine (µmol/l)*	83.00 ± 18.42
*Cholesterol (mmol/l)*	5.22 ± 1.10
– HDL	1.21 ± 0.46
– Triglyceride	2.05 ± 1.01
– LDL	3.04 ± 1.02
Procedure characteristics	
*Average days between CCTA and FFR (median (IQR))*	28.00 (14.75–59.75)
*Calcium score (median (IQR))*	317.0 (112.5–725.0)
< 100	13 (21.3)
100–400	19 (32.1)
> 400	23 (37.7)
Missing	6 (9.8)
*CCTA*	
< 50% diameter stenosis	39 (44.3)
50–69% diameter stenosis	15 (17.0)
≥ 70% diameter stenosis	34 (38.6)
*CT-FFR*	0.78 ± 0.09
*FFR*	0.80 ± 0.08

*BMI* body mass index, *CAD* coronary artery disease, *CCTA* coronary computed tomography angiography, *FFR* fractional flow reserve, *CT-FFR* computed tomography fractional flow reserve, *HDL* high-density lipoprotein, *IDDM* insulin-dependent diabetes mellitus, *IQR* interquartile range, *LDL* low-density lipoprotein, *NIDDM* non-insulin-dependent diabetes mellitus, *SD* standard deviation

**Table 2:** Diagnostic performance of computed tomography-derived fractional flow reserve (*CT-FFR*) and coronary computed tomography angiography (*CCTA*) per vessel and per patient. The diagnostic performance of CT-FFR and CCTA with FFR as reference standard. FFR ≤ 0.80, CT-FFR ≤ 0.80 and CCTA ≥ 50% are used as diagnostic cut-off values

	**CT-FFR per vessel**			**CT-FFR per patient**			**CCTA ≥ 50% per vessel**			**CCTA ≥ 50% per patient**		
*TP*	31 (35.2%)			30 (49.2%)			32 (36.4%)			30 (49.2%)		
*TN*	44 (50.0%)			21 (34.4%)			33 (37.5%)			15 (24.6%)		
*FP*	10 (11.4%)			8 (13.1%)			21 (23.9%)			14 (23.0%)		
*FN*	3 (3.4%)			2 (3.3%)			2 (2.3%)			2 (3.3%)		
	**Estimate (%)**	**95% CI (%)**		**Estimate (%)**	**95% CI (%)**		**Estimate (%)**	**95% CI (%)**		**Estimate (%)**	**95% CI (%)**	
*Sensitivity*	91.2	77.0	97.0	93.8	79.9	98.3	94.1	80.9	98.4	93.8	79.9	98.3
*Specificity*	81.5	69.2	89.6	72.4	54.3	85.3	61.1	47.8	73.0	51.7	34.4	68.6
*NPV*	93.6	82.8	97.8	91.3	73.2	97.6	94.3	81.4	98.4	88.2	65.7	96.7
*PPV*	75.6	60.7	86.2	78.9	63.7	88.9	60.4	46.9	72.4	68.2	53.4	80.0
*Accuracy*	85.2	76.3	91.2	83.6	72.4	90.8	73.9	63.8	81.9	73.8	61.6	83.2

*95% CI* 95% confidence interval, *FN* false-negative, *FP* false-positive, *NPV* negative predictive value, *PPV* positive predictive value, *TN* true-negative, *TP* true-positive

## Discussion

In this cross-sectional single-centre study, we determined the diagnostic performance of an on-site lumped-parameter CT-FFR algorithm and compared this to CCTA. CT-FFR showed good diagnostic accuracy in identifying haemodynamically relevant stenosis in patients with complaints of stable chest pain and a low or intermediate probability of CAD using the reference standard FFR. Moreover, it showed improved accuracy compared to CCTA alone. This study demonstrated the feasibility of the on-site CT-FFR approach for patients with stable CAD referred for ICA. Since the diagnostic performance of CT-FFR was superior to that of CCTA, it seems an effective step after a positive or inconclusive CCTA procedure.

The only commercially available CT-FFR algorithm (HeartFlow) is approved by the Food and Drug Administration and included in the National Institute for Health and Care Excellence (NICE) guidelines.

A meta-analysis by Celeng et al. described a pooled sensitivity on a per-vessel basis of 85% (CI: 81–90) and a pooled specificity of 73% (CI: 61–82), indicating that CT-FFR has improved diagnostic accuracy compared with CCTA [7]. The sensitivity amongst all studies is within a small range compared to specificity, which varies greatly between studies. The prospective multicentre trial PLATFORM demonstrated that a CT-FFR-guided strategy can reduce up to 61% of the planned ICA procedures compared to usual care [10]. Besides, it is demonstrated that the use of CT-FFR is associated with similar clinical outcomes and quality of life over 1 year of follow-up [24]. Moreover, a CT-FFR-guided strategy leads to a decrease in costs compared to usual care. One of the disadvantages of off-site CT-FFR is the percentage of CCTA images rejected by the CT-FFR core laboratory. In the clinical trials performed so far, rejection rates of between 2.9% and 33% were reported, mainly due to poor image quality and artefacts [8, 11, 12, 26]. Recently, in 10,621 consecutive patients who underwent HeartFlow FFR_CT_ for clinical analysis, Pontone et al. reported a rejection rate of 8.4% [27].

Multiple vendors have developed algorithms that can be used on-site to assess stable CAD [28]. Siemens (cFFR version 1.4) developed the software that has been evaluated most often. The sensitivity ranges between 82% and 86%, while the specificity range is 63–83% [17–21, 29]. A sensitivity of 83% and a specificity of 84–88% were reported using the CT-FFR algorithm of Toshiba (Toshiba Medical Systems, Tokyo, Japan), which is not commercially available [30, 31]. A third on-site CT-FFR algorithm was developed by Fujimoto et al. and has a high sensitivity (91%) and a moderate specificity (78%) [22]. The CT-FFR algorithm evaluated in the current article has been tested previously in two studies. A sensitivity of 91%, a specificity of 72% and an accuracy of 78% were reported by Donnelly et al. [13], whereas van Hamersvelt et al. [15] found a sensitivity of 89%, specificity of 78% and an accuracy of 83%, both comparable to the findings in the current study. The workflow of all on-site CT-FFR techniques seems to be similar, while differences can be found in the underlying algorithm and specific boundary conditions. The assumptions made lead to estimates of the diagnostic value that slightly differ, and lead to pooled estimates of sensitivity of 84% (80%; 88%) and specificity of 80% (73%; 86%) [7]. The sensitivity found in this study is higher than the pooled sensitivity (91.2%) and has a comparable specificity (81.4%). Besides the CT-FFR algorithms, prototypes of deep-learning analysis of the coronary arteries to identify patients with functionally significant CAD have been developed [14, 16, 32].

The widespread implementation of CT-FFR in the current work-up for patients with suspected stable CAD might be feasible. CT-FFR provides additional diagnostic information compared to the existing pathways, is easy and fast to use, reproducible, and can potentially be cost-effective. CT-FFR seems to add value after positive or inconclusive CCTA, especially since it does not require additional testing, radiation or contrast medium. On-site CT-FFR can easily be integrated in the workflow; however it requires approximately 20 min additional operator time, since the segmentation of the coronary centrelines is performed semi-automatically. This depends heavily on the scan quality and the amount of calcification present. Reduction of the operator time by improvements in semi-automatic centreline extraction will lead to a decrease in operator time and improve the workflow.

There are limitations to our study. The retrospective nature of the data collection induces selection bias towards a high coronary disease burden. Most patients underwent invasive FFR if clinically indicated, which results in a higher pre-test probability. In general, this bias leads to a too high sensitivity and a too low specificity estimate [33]. The level of performance of CT-FFR in a standard population with a normal coronary disease prevalence is unknown. The reference standard of this study, FFR, is a measure of coronary pressure as derivative for coronary blood flow. FFR and coronary flow are highly correlated, but not identical. Moreover, FFR itself varies between repeated measurements [34]. Another limitation is that most CT scans (79%) are acquired using old 64-slice scanners and are therefore of relatively low quality. Since diagnostic accuracy depends on imaging quality, we expect an increase in diagnostic performance by using 256-slice systems. Due to the imaging quality of the current scans, vessel annotation was challenging in some patients. Major manual adjustments of the centrelines and lumen were made, which has an impact on reproducibility.

The correlation between CT-FFR and invasive FFR is imperfect, mainly because CT-FFR is dependent on accurate 3D coronary models. Technical and acquisition features that could impact the correlation negatively are image artefacts caused by cardiac and respiratory motion, low contrast, tachycardia or arrhythmia leading to a stair-step artefact, phase misregistration and blooming [35]. Other factors that could explain the intermediate correlation are the multiple assumptions made concerning boundary conditions (inlets, outlets and vessel walls) [7, 35]. Differences in micro-vascularisation and distal outlet conditions are not incorporated [36].

In conclusion, the CT-FFR algorithm enabled assessment of the functional characteristics of CAD in addition to the anatomical interpretation obtained on CCTA. CT-FFR achieved a good diagnostic performance and its on-site use is feasible. It improves diagnostic accuracy compared to CCTA without additional testing and therefore has the potential to be implemented in the current clinical work-up for diagnosing stable CAD.
